# Increased IKKα Expression in the Basal Layer of the Epidermis of Transgenic Mice Enhances the Malignant Potential of Skin Tumors

**DOI:** 10.1371/journal.pone.0021984

**Published:** 2011-07-06

**Authors:** Josefa P. Alameda, Rodolfo Moreno-Maldonado, M. Jesús Fernández-Aceñero, Manuel Navarro, Angustias Page, José L. Jorcano, Ana Bravo, Ángel Ramírez, M. Llanos Casanova

**Affiliations:** 1 Division of Epithelial Biomedicine, CIEMAT, Madrid, Spain; 2 Department of Pathology, Fundación Jiménez Díaz, Madrid, Spain; 3 Department of Veterinary Clinical Sciences, Veterinary Faculty, University of Santiago de Compostela, Lugo, Spain; Université de Technologie de Compiègne, France

## Abstract

Non-melanoma skin cancer is the most frequent type of cancer in humans. In this study we demonstrate that elevated IKKα expression in murine epidermis increases the malignancy potential of skin tumors. We describe the generation of transgenic mice overexpressing IKKα in the basal, proliferative layer of the epidermis and in the outer root sheath of hair follicles. The epidermis of K5-IKKα transgenic animals shows several alterations such as hyperproliferation, mislocalized expression of integrin-α6 and downregulation of the tumor suppressor maspin. Treatment of the back skin of mice with the mitogenic agent 12-*O*-tetradecanoylphorbol-13-acetate causes in transgenic mice the appearance of different preneoplastic changes such as epidermal atypia with loss of cell polarity and altered epidermal tissue architecture, while in wild type littermates this treatment only leads to the development of benign epidermal hyperplasia. Moreover, in skin carcinogenesis assays, transgenic mice carrying active Ha-*ras* (K5-IKKα-Tg.AC mice) develop invasive tumors, instead of the benign papillomas arising in wild type-Tg-AC mice also bearing an active Ha-*ras*. Therefore we provide evidence for a tumor promoter role of IKKα in skin cancer, similarly to what occurs in other neoplasias, including hepatocarcinomas and breast, prostate and colorectal cancer. The altered expression of cyclin D1, maspin and integrin-α6 in skin of transgenic mice provides, at least in part, the molecular bases for the increased malignant potential found in the K5-IKKα skin tumors.

## Introduction

Keratinocytes of the basal layer of the epidermis are mitotic, providing new cells to replace those that are shed. After moving to the suprabasal layers, the cells gradually differentiate and give rise to the cornified layer at the surface of the skin that protects the internal organs. Therefore, a balance between keratinocyte proliferation and differentiation is required to maintain epidermal homeostasis. IKKα (IκB kinase α) is a fundamental component of the IKK complex that regulates the NF-κB signalling pathway [1]–[3]. IKKα has a fundamental role in regulating keratinocyte proliferation and differentiation [4]–[8]. The epidermis of IKKα−/− newborn mice lacks a terminally differentiated cornified layer and exhibits marked thickening [6]–[8]. Reintroduction of IKKα or a kinase-inactive mutant IKKα induces terminal differentiation of keratinocyte and represses hyperproliferation [9], [10]. This demonstrates that IKKα is necessary for epidermal differentiation independently of its kinase activity [9]. We have described that IKKα increases the differentiation of human keratinocytes by a mechanism dependent on E-cadherin [5]. Other adhesion molecules such as claudin-23, occludin and desmoglein 3 have also been found to be regulated by IKKα and to play a role in epidermal terminal differentiation and skin barrier function [11].

Non melanoma skin cancer (NMSC) is the most common malignancy in humans: BCCs (basal cell carcinomas) and SCCs (squamous cell carcinomas) represent the vast majority of the tumors diagnosed. The incidence of both benign and malignant NMSC has been rising at an alarming rate for the past several years. The role of IKKα in cancer development remains controversial: while it has been suggested that it functions as a tumor suppressor in skin cancer [4], [12], [13], there are also evidences that support a role of IKKα as promoter of cancer progression and metastasis in different types of neoplasias such as breast cancer [14], hepatocarcinomas [15], prostate cancer [16], [17] and colorectal cancer [18], [19]. Indeed, we have found, in xenograft assays, an increase in malignancy of skin tumors over-expressing IKKα [5].

In the last years it has been found that IKKα regulates the expression of molecules implicated in cancer development such as the tumor suppressor maspin (mammary serine protease inhibitor), twist, and adhesion proteins. It inhibits cellular motility, invasiveness and angiogenesis, and provides sensitivity to apoptosis in tumor cells [20], [21]. Maspin expression predicts a better prognosis in different types of cancers: breast [22], [23], prostate [24], [25], colon [26], oral squamous cell carcinoma [27], lung [28], larynx [29], malignant melanoma [30] and ovarian cancer [31], although recently it has been reported that it might act as tumor promoter in colorectal or pancreatic cancers [32], [33]. Maspin also acts as a suppressor of metastasis in different types of cancer such as prostate, liver and breast [17], [34]. Interestingly, IKKα inhibits maspin expression and promotes cell metastasis in prostate cancer and hepatocarcinomas [15], [17], [35]. Twist is a basic-helix-loop-helix (bHLH) protein known to be essential during the embryogenesis which also plays an important role as mediator of EMT (epidermal mesenchymal transition) during tumor progression [36]. Twist is overexpressed in a large set of human and murine tumors including sarcomas, melanomas, gliomas and neuroblastomas [37], [38] being the reactivation of Twist indicative of poor prognosis. Interestingly, it has been reported that IKKα null embryos express reduced levels of Twist [7] which suggest a positive regulation of Twist expression by IKKα. The altered expression of adhesion molecules has been related to tumor development, included skin cancer, i.e., overexpression of the adhesion protein integrin-α6 in the basal layer of epidermis and hair follicles has been reported to cause malignization of skin tumors in transgenic mice [39]. Moreover, α6β4 integrin expression in suprabasal strata serves as an early predictive marker to identify benign squamous tumors at high risk of malignant progression [40].

In this work we have analyzed the effect of increased levels of IKKα expression in the basal layer of the epidermis of transgenic (Tg) mice (K5-IKKα mice), and its repercussion in *in vivo* skin carcinogenesis. We have found that K5-IKKα mice exhibit in epidermis several alterations, such as increased proliferation, suprabasal integrin-α6 expression and downregulation of the tumor suppressor maspin. In line with these alterations, the application of a mitogenic agent, i.e. TPA in the back skin of mice leads in transgenic K5-IKKα mice to the appearance of preneoplastic features such as epidermal atypia with loss of cell polarity and altered epidermal tissue architecture, while in wild type (WT) littermates this treatment only leads to the development of benign epidermal hyperplasia. Moreover, in carcinogenesis experiments, tumors developed in transgenic mice carrying active Ha-*ras* (Tg.AC mice) are invasive tumors, in sharp contrast with the benign tumors originated in WT animals (also bearing an active Ha-*ras*). In addition to integrin-α6 suprabasal overexpression, tumors developed in the K5-IKKα mice show reactivation of the expression of Twist.

## Materials and Methods

### Ethics Statement

All experimental procedures were performed according to European and Spanish laws and regulations (European Convention ETS 123 on the use and protection of vertebrate mammals used in experimentation and other scientific purposes; Spanish R.D 1201/2005 of the Ministry of Agricultural, Food and Fisheries on the protection and use of animals in scientific research) and approved by the our institution's ethics committee. Approval ID: BME 3/06; BME 1/09 and BME 2–10 by the CIEMAT Institution's ethics commitee.

### Generation of Tg mice

**Figure 1:**
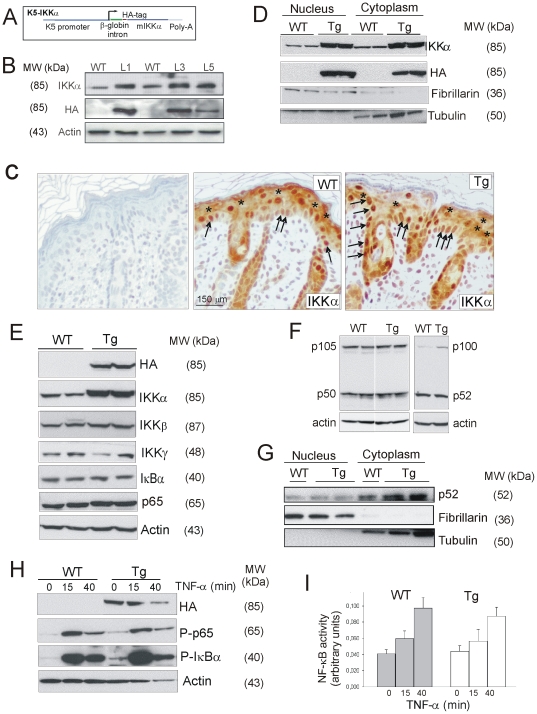
Characterization of K5-IKKα transgenic mice. (**A**) Recombinant DNA construct employed to generate transgenic mice. (**B**) Western blots showing IKKα and HA expression in WT and 3 different transgenic lines (L1, L3 and L5). Actin was used as a loading control. (**C**) Expression of the IKKα protein in back skin of 3-day-old mice. Immunostaining with the Sc-7182 antibody is showed (middle and right panels); similar results where obtained with the IMG-90454 anti IKKα antibody (not shown). Control without primary antibody (only with secondary biotinylated anti-rabbit antibody) is shown as control of specificity of the immunostaining against IKKα (left panel). Arrows show the expression of IKKα in basal epidermal keratinocytes and hair follicles. Asterisks indicate cytoplasmic expression of IKKα. (**D**) IKKα and HA detection in nuclear and cytoplasmic protein extracts from 5 day-old WT and Tg mice. Samples from two different mice of the line L1 and two different control littermates were loaded. Fibrillarin and tubulin show loading control and purity of the extracts. (**E**) Western blots analysis of the expression of different members of the NF-κB pathway in skin of WT and K5-IKKα transgenic mice. Total proteins from backskin of two different mice of the line L1 and two different control littermates were loaded. No differences were found between mice of both genotypes. (**F**) Western blot showing p100 and p105 processing in skin of WT and Tg mice. No differences were found. (**G**) Western blot showing the nuclear localization of p52 in skin of WT and Tg mice. No differences were found. (**H**) p65 and IκBα phosphorylation kinetics; actin: loading control. Newborn WT and Tg mice were injected with TNF-α and analyzed at the indicated times. (**I**) NF-κB activity in skin of non-stimulated and TNF-α injected mice for the indicated times. Experiments were repeated 3 times with similar results.

HA-tagged murine IKKα [5] was placed under the control of a 5.2 kb 5′-upstream fragment of bovine K5 promoter and a rabbit β-globin intron (Figure 1A). Tg mice were generated by microinjection of this construct into B6D2F2 embryos using standard techniques and Tg lines were maintained by crossing with B6D2F1 mice. Mice were genotyped by PCR analysis of tail genomic DNA using primers specific for the rabbit β-globin intron. Wild type non-transgenic littermates were used as control animals.

### Western Blotting

Total or nuclear and cytoplasmic protein extracts (40 µg) were subjected to SDS/PAGE. The separated proteins were transferred to nitrocellulose membranes (Amersham, Arlington Heights, IL) and probed with antibodies against IKKα, IKKβ, IKKγ (IMGENEX, San Diego, CA, USA); HA epitope (Covance, California, USA); IκBα, p105/p50, p65, maspin, actin, fibrillarin, EGFR (Santa Cruz Biotechnology, Inc. Europe); p100/p52 (Abcam, Cambridge, UK); P-p65, P-IκBα (Cell Signaling Technology, Danvers, MA, USA); Cyclin D1 (NeoMarkers, Fremont, CA, USA); P-tyrosine (4G10; Upstate, NY, USA), and tubulin (Sigma, Saint-Louis, Missouri, USA). In all cases samples were subjected to luminography with the Supersignal West Pico Chemiluminescent Substrate (Pierce Biotechnology, Inc., Illinois, USA). Densitometric analysis of the blots was performed using the Molecular Analyst software package (Bio-Rad Laboratories Inc., Hercules, California, USA).

### Histology and Immunohistochemistry

Skin and tumors were fixed in 10% buffered formalin or in 70% ethanol and embedded in paraffin. Sections were stained with H&E for histopathological evaluation or used for immunostaining using primary antibodies raised against IKKα (sc-7182, Santa Cruz Biotechnology, Inc. Europe; IMG-90454, IMGENEX), keratin K1, K5, K10, involucrin, loricrin, filaggrin (Covance); keratin K13, Twist (Abcam); integrin-α6 (CD49f; BD Pharmingen), BrdU (Roche, Mannheim, Germany) and Cleaved Caspase-3 (Cell Signaling Technology, Danvers, MA, USA). Sections were incubated with a biotinylated anti-mouse, anti-rat or anti-rabbit antibody, and then with streptavidin conjugated to horseradish peroxidase (DAKO A/S, Glostrp, Denmark). Antibody localization was determined using 3,3-diaminobenzidine (DAB) in PBS (Vector Laboratories; Burlingame, CA, USA).

### BrdU treatment

Mice received an intraperitoneal injection of BrdU 120 mg/kg body weight 1 h before sample harvesting.

### TNF-α *in vivo* treatment

3 days-old mice were subcutaneously injected with 20 µg/Kg of human TNF-α (Sigma) or with PBS (control). After the indicated times mice were sacrificed, skin samples removed and proteins extracted.

### NF-κB activity assay

NF-κB DNA binding assays was determined by the NF-κB p50/p65 EZ-TFA Transcription Factor Assay (Millipore, Massachusetts, USA) following manufacturer's instructions [41]. Briefly, protein extracts from WT and Tg skins (12.5 µg) were mixed with a double stranded biotinylated oligonucleotide containing the consensus sequence for NF-κB binding. In this way, activated NF-κB (active p65) contained in the extracts binds to its consensus sequence. This mixture is transferred to a streptavidin coated plate and the bound NF-κB subunit, p65, is detected with a specific primary antibody. An HRP-conjugated secondary antibody is then used for detection and provides sensitive colorimetric detection that can be read in a spectrophotometric plate reader (Genios Pro, TECAN, Madrid, Spain; XFluor4Version V4.50).

### TPA treatment

To induce epidermal hyperplasia, six K5-IKKα 8-week-old mice and six WT mice of the same age (8 weeks) were used. Shaved dorsal skins were treated twice a week with 5 µg of 12-*O*-tetradecanoylphorbol-13-acetate (TPA; Sigma) for 3 weeks. Mice were sacrificed 24 hours after the last application.

### Carcinogenesis assays

In the DMBA/TPA protocol, both, K5-IKKα and WT 9-week-old mice (9 animals respectively) were initiated with a single dose of 200 nmol of 7,12-dimethyl-benz[a]anthracene (DMBA) (Sigma) on shaved dorsal skin. Two weeks later, tumor growth was promoted by treating with 5 µg of TPA twice a week per 20 weeks. In the carcinogenesis experiments in Tg.AC background, female homozygous v-Ha-*ras* transgenic Tg.AC mice [42] were mated with K5-IKKα males. Double transgenic K5-IKKα-TgAC and WT-TgAC 9-week-old mice (11 animals respectively) were treated twice weekly with topical applications of 5 µg of TPA in 200 µl acetone for 7 weeks according to standard protocols. Experimental procedures were performed according to European and Spanish laws on experimental animal protection.

### Statistics

Statistical significance of data was assessed using the t-test and the Mann-Whitney (Wilcoxon) W test.

## Results

### Increased expression of IKKα in basal keratinocytes of K5-IKKα transgenic mice

We generated the K5-IKKα transgenic mice overexpressing a mouse IKKα cDNA tagged with an epitope from hemagglutinin A (HA) (Figure 1A). The keratin 5 (K5)-derived sequences included in this construct drive transgene expression to the basal cells of the epidermis and outer root sheath (ORS) of hair follicles, as well as to internal stratified epithelia [43], [44]. K5-IKKα transgenic mice developed normally and showed no obvious alterations. Immunoblotting analysis using specific antibodies against IKKα revealed increased expression of IKKα in the skin of different K5-IKKα Tg lines (Figure 1B). HA epitope was detected in skin of K5-IKKα-Tg mice but not in skin of WT mice (Figure 1B). L1 and L3 were the highest IKKα expressing lines and similar results were obtained in the analysis of both of them, therefore we performed most of the following experiments in line L1. The immunohistochemical staining of IKKα in back skin of WT and Tg mice showed higher IKKα staining in the basal layer of the epidermis and the ORS of hair follicles of K5-IKKα-Tg mice than in WT littermates (Figure 1C). IKKα immunostaining was performed with two different antibodies (see Mat and Met section) and repetitively IKKα expression was detected both in the cytoplasm and in the nucleus of suprabasal and basal keratinocytes in WT and Tg animals (Figure 1C). WB analysis confirmed this nuclear and cytoplasmic localization of IKKα and also its overexpression in K5-IKKα-Tg skin (Figure 1D). HA immunostaining gave similar results (data not shown).

### Classical and non-canonical NF-κB activation pathways are not affected in the K5-IKKα-Tg mice

The state of the NF-κB pathway in WT and K5-IKKα mice was analyzed. In agreement with other studies [8], we found that changes in IKKα expression do not alter the expression of other members of the NF-κB pathway such as IKKβ, IKKγ, IκBα and p65 (Figure 1E). In addition no differences were found in p100 or p105 processing, nor in nuclear p52 localization in WT and Tg mice skin (Figure 1F, G), indicating that the non-canonical pathway of NF-κB does not seem altered by IKKα overexpression. Treatment of newborn mice with tumor necrosis factor α (TNF-α) led to similar kinetics and extension of p65 and IκBα phosphorylation in WT and Tg mice (Figure 1H). In addition, we analyzed the NF-κB pathway by measuring NF-κB binding activity in protein extracts from skins of mice of each genotype and found no differences in non-stimulated skin as well as after TNF-α subcutaneous injection of WT and K5-IKKα mice (Figure 1I). Together, these results indicate that the classical IKK/NF-κB pathway is not modified in K5-IKKα Tg mice, and are in accordance with our previous data in human HaCaT keratinocytes [5] where the overexpression of IKKα did not alter this signalling pathway.

### Delocalized integrin-α6 suprabasal expression, increased proliferation and maspin inhibition in epidermis of K5-IKKα-Tg mice

**Figure 2:**
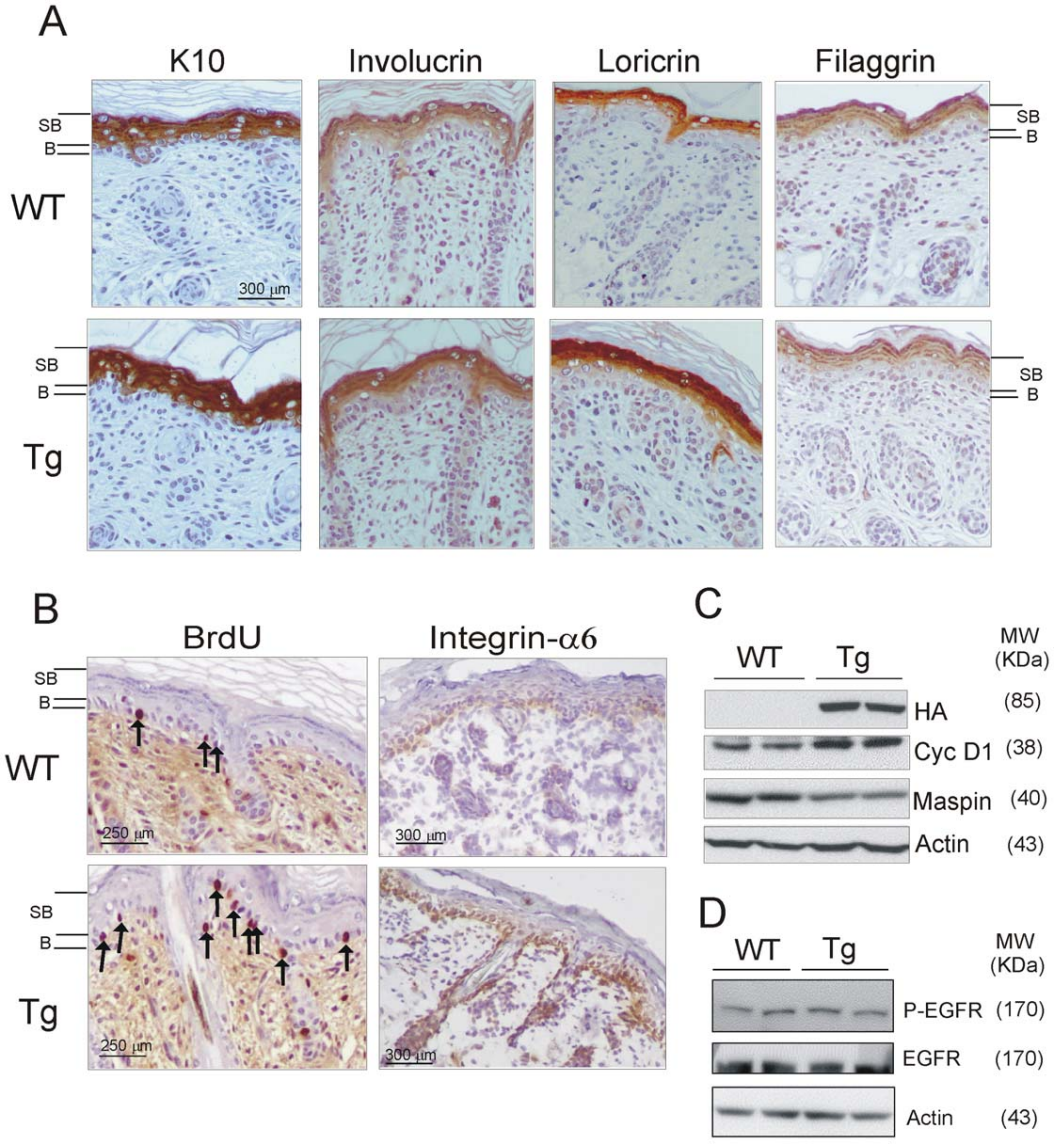
Alterations found in back skin of K5-IKKα-Tg mice. (**A**) Immunohistochemical analysis of epidermal differentiation markers in back skin of 3-day-old WT and K5-IKKα mice. No differences were found between mice of both genotypes. B, SB, basal and suprabasal keratinocyte layers respectively. (**B**) Images show the increased number of basal keratinocytes that incorporate BrdU in epidermis of Tg mice. Integrin α6 is expressed in basal as well as suprabasal keratinocytes in skin of Tg mice. (**C; D**) Analysis of cyclin D1 (Cyc D1), maspin and EGFR protein levels as well as EGFR phosphorylation (P-EGFR) in total protein extracts of skin of WT and K5-IKKα-Tg mice. Total proteins from backskin of two different mice of the line L1 and two different control littermates were loaded.

As IKKα has an essential role in epidermal morphogenesis and differentiation, we analyzed early and late differentiation markers of the epidermis such as K1, involucrin, loricrin, and filaggrin (Figure 2A). No appreciable differences were found between WT and K5-IKKα-Tg mice, suggesting that no alterations of the normal epidermal differentiation program occur in these animals. The expression of K5 and K6 also was unaltered (data not shown). We also analyzed the proliferative capability of WT and K5-IKKα-Tg mice skin by measuring BrdU incorporation. We observed increased BrdU-positive cells in the basal layer of the epidermis of K5-IKKα mice (11.4±2.7% in Tg mice *versus* 7.6±1.7% in WT mice; n = 6; P<0.05) (Figure 2B). Cyclin D1, another marker of proliferation was also increased in the skin of Tg mice (Figure 2C). In an attempt to discover a possible cause for the increased proliferation observed in skin of transgenic mice, we measured EGFR expression, as this is an important factor for keratinocyte proliferation [45]; however, we found no differences in EGFR levels nor in EGFR phosphorylation in skin of WT and K5-IKKα Tg mice (Figure 2D). Another reason for the increase in the number of proliferating cells in the skin of Tg mice could be the altered expression of adhesion molecules; in particular, the suprabasal expression of integrin-α6 has been found to be associated with hyperproliferative conditions in dermal equivalent cultures of keratinocytes [46]. We therefore analyzed the expression of integrin-α6 in the back skin of WT and Tg newborn mice and found that while WT mice skin exhibit integrin-α6 expression restricted to the basal layer of the epidermis, K5-IKKα-Tg mice showed increased and delocalized integrin-α6 expression in basal as well as suprabasal keratinocytes (Figure 2B). As these parameters (i.e. increased proliferation and altered expression of integrin-α6 in epidermis) are signals that may precede a malignization process [39], we checked the expression of maspin, a tumor suppressor known to be negatively regulated by IKKα. We found by Western blotting of total skin extracts that maspin was downregulated in the skin of K5-IKKα-Tg mice (Figure 2C), in agreement with other studies where IKKα overexpression leads to the inhibition of maspin expression [15], [17].

### Epidermal atypia of the epidermis in K5-IKKα transgenic mice treated with TPA

**Figure 3:**
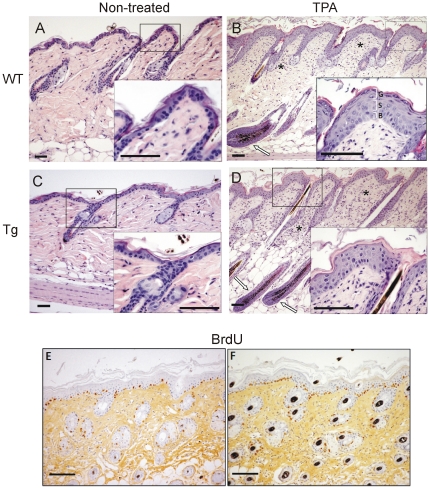
Atypical epidermal hyperplasia in skin of K5-IKKα-Tg mice subjected to TPA treatment. (**A–D**) Representative paraffin-sections of skin from WT (**A–B**) and K5-IKKα-Tg mice (**C–D**) were stained with hematoxilin/eosin. (**A, C**) Non-treated skins; (**B, D**) TPA treated skins. (**B**) Hyperplastic epidermis induced by topical application of TPA in WT mice. White arrow indicates a hair follicle in the anagen phase. (Inset in B) B: *stratum basale*, S: *stratum spinosum*, G: *stratum granulosum*. Conservation of squamous differentiation is clear in WT epidermis where marked hyperplasia of *stratum spinosum* (acantosis) and *stratum granulosum* (hipergranulosis) are observed as common facts of typical epidermal hyperplasia. (**D**) Hyperplasia and epidermal atypia with loss of cellular architecture in TPA-treated Tg skin. White arrows indicate hair follicles in the anagen phase. (Inset in D) Epidermal hyperplasia in K5-IKKα-Tg mice shows an atypical proliferating epithelium with all strata conformed by keratinocytes with round, central nuclei highly basophilic (pyknosis). The *stratum granulosum* of flattened cells with cytoplasm filled with keratohyaline granules were not formed. Asterisks in B, D points to regions of the lichenoid inflammation with diffuse infiltrates of mononuclear cells found in skin of both WT and Tg mice after TPA treatment. (**E, F**) BrdU incorporation in skins from WT (**E**) and Tg (**F**) mice treated with TPA. (**A–F**): scale bars = 50 µm.

To analyze whether the alterations found in the skin of transgenic K5-IKKα mice could predispose these mice to the development of more severe lesions when subjected to skin insult, we applied multiple doses of the mitogenic agent, TPA, to the back skin of WT and Tg mice in the second telogen phase of the hair follicles. As expected, 3 weeks of treatment provoked in WT animals the entry of hair follicles into the anagen phase as well as the development of epidermal hyperplasia (7 to 10 layers of epidermal keratinocytes *versus* 2 to 4 layers observed in non-treated epidermis of adult mice; Figure 3 compare A and B). We observed that the hyperplasia was due to an increase both in the *stratum spinosum* and *granulosum* (see inset in B). This hyperplasia exhibit the typical increase in epidermal thickness, with keratinocytes of the *stratum basale* containing nuclei perpendicular to the basal membrane; keratinocytes of the *stratum spinosum* with round, central nuclei; and keratinocytes of the *stratum granulosum* showing progressive flattening of the cells, in parallel to the basal membrane, with smaller nucleus and scarce cytoplasm filled with keratohyaline granules. In Tg mice, TPA application also induces hair follicle anagen phase and epidermal hyperplasia (Figure 3 compare C and D). However, in these mice the hyperplasia was associated with epidermal atypia, characterized by a disorganized architecture of the different keratinocyte layers where cells lose their polarity and normal differentiation; it was not possible to distinguish the *stratum basale, spinosum* and *granulosum* because most keratinocytes appeared as round cells with central nucleus and there was no sign of terminal differentiation (i.e. lack of keratohyaline granules in the upper suprabasal layers) (see inset in Figure 3D and compare with inset in B). There was also a great heterogeneity in the nuclear staining of keratinocytes from Tg-TPA-treated mice showing many condensed, highly basophilic pyknotic nuclei. Elevated rates of keratinocyte proliferation (measured as BrdU incorporation) were observed in the epidermis of both WT and Tg mice treated with TPA (Figure 3 E, F), being significantly higher the number of proliferating cells in the K5-IKKα mice (Figure 3F) compared with WT siblings (Figure 3E) (labelling index, Tg = 29.1±3.7; WT = 18.8±4.0; n = 6; P<0.05). The treatment with TPA in addition to a mitogenic effect also provokes an inflammatory response. We have analyzed this effect in skin of WT and transgenic mice and have found a similar response in both cases i.e. a mild lichenoid inflammation with diffuse infiltrates of mononuclear cells in the superficial dermis (Figure 3B and D asterisks).

### Enhanced malignant potential of tumors developed in K5-IKKα transgenic mice

We performed two different approaches to investigate the susceptibility of K5-IKKα mice to develop skin cancer. We initiated WT and K5-IKKα mice with a single dose of DMBA followed by TPA application in a conventional two-stage (DMBA/TPA) tumorigenesis protocol. DMBA activates Ha-*ras* to initiate skin tumors [47]. During the course of the experiment we did not observe significant differences in the number of tumors between WT and Tg mice (Figure S1A). Tumors were traced until week 27, when they were collected. The average tumor size was also similar in both groups (data not shown). In the other approach we performed TPA treatments in F1 crosses of K5-IKKα mice with the Tg.AC mice strain, carrying an activated Ha-*ras* transgene that triggers the classic initiation event [42], [44], [48]. Both groups of mice (WT-TgAC and K5-IKKα-TgAC) developed papillomas with a similar latency period (5–7 weeks, Figure S1B). The percentage of animals that developed tumors, as well as the tumor multiplicity, was similar in both genotypes. From week 17 on, the number of tumors in WT mice in both approaches was reduced, due probably to a higher regression rate (Figure S1A, B).

**Figure 4:**
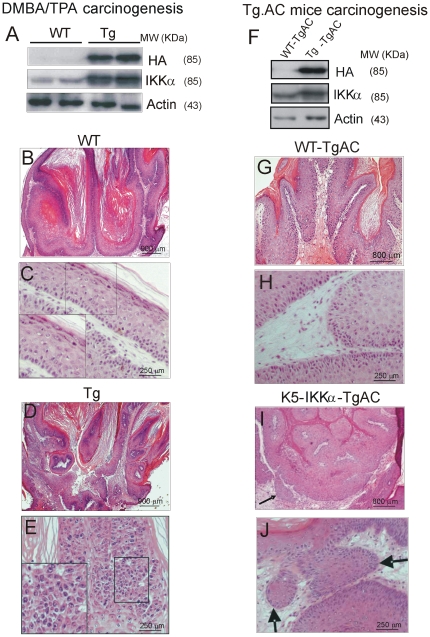
Tumors from K5-IKKα transgenic mice show increased malignancy. (**A, E**) Tumors developed in WT and Tg mice in the DMBA/TPA skin carcinogenesis assays. (**A**) Western blot showing the expression of the transgene in the tumors. Total protein extratcs from two different tumors developed in transgenic mice of the line L1 and proteins from two different control mice were loaded. (**B**–**E**) Histology of tumors arising in mice subjected to the DMBA/TPA approach. Note the epithelial atypia areas in Tg tumors (inbox in **E**) versus the well conserved tissue architecture in WT tumors (**C**). (**F**–**J**) Tumorigenesis in Tg.AC mice. (F) Western blot showing the expression of the transgene in the tumors. Total protein extratcs were loaded. (**G, H**) Development of benign tumors in WT-TgAC mice versus invasive tumors K5-IKKα-TgAC mice (**I, J**)**.** (**J**) High magnification showing the rupture of the basement membrane and invasion of tumor epidermal cells into the stroma of transgenic tumors. Arrows in (**I, J**): focal invasion areas.

**Figure 5:**
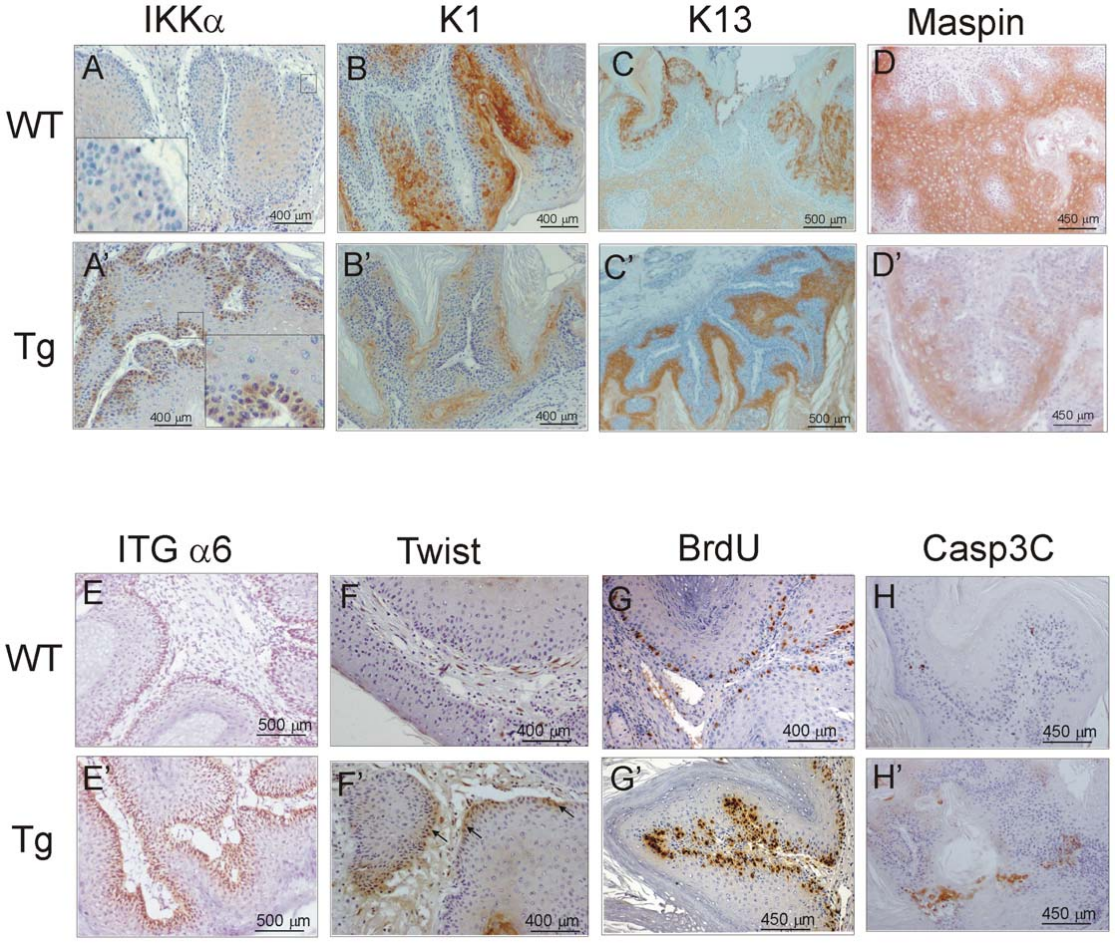
Immunohistochemical analysis of tumor markers in WT and K5-IKKα DMBA/TPA tumors. (**A, A**') IKKα is scarcely and diffusely expressed in the suprabasal cells of WT tumors while it is strongly expressed in K5-IKKα tumors, mainly in the basal cells, although it is also detected in the suprabasal cells. (**B, B**') Elevated expression of K1 is detected in WT tumors while Tg tumors exhibit scarce K1 staining. (**C, C**') K13 immunostaining shows that it is weakly expressed in WT tumors versus the strong expression detected in Tg tumors. (**D, D**') Lower staining of Maspin in K5-IKKα tumors. (**E, E**') Strong and delocalized integrin α6 staining is detected in K5-IKKα tumors while WT tumors show integrin α6 basal expression. (**F, F**') Twist is expressed in Tg tumors while it is not detected in WT tumors. (**G, G**') Higher proliferation rate (measured as BrdU incorporation) is detected in Tg tumors (both in the basal and suprabasal layers) compared to the lower proliferating cells found in the basal layer of WT tumors. (**H, H**') Increased Caspase 3 cleaved (Casp3C) staining in K5-IKKα tumors compared to that in WT tumors.

We checked by Western blot analysis that the tumors developed in Tg mice in both carcinogenesis protocols express higher levels of IKKα than WT tumors and also express the HA-tag (Figure 4A, F). The histological study showed important differences between tumors developed in WT and K5-IKKα mice in the two approaches. Thus, while tumors arising in WT mice in the DMBA/TPA tumorigenesis were benign papillomas with a well conserved differentiation pattern of the epidermis (Figure 4B, C), those developed by K5-IKKα mice exhibited extended areas of epithelial atypia (Figure 4D, E), indicating a higher malignant potential. These lesions resemble those found in the epidermis of Tg mice treated with TPA (Figure 3D) although of higher aggressive potential. The tumorigenesis assays in Tg.AC mice showed that tumors from WT-TgAC animals were benign papillomas (Figure 4G, H), while tumors from K5-IKKα-TgAC mice showed areas of focal invasion, i.e., microinvasive infiltration, indicating a higher degree of malignant progression (Figure 4I, J). The immunohistochemical analysis of tumors developed in WT mice in both types of carcinogenesis protocols showed a diffuse expression of IKKα in the suprabasal cells (Figure 5A). By contrast, a higher staining for IKKα was detected in the K5-IKKα tumors obtained by both approaches (Figure 5A'), in accordance with the results of the Western blot analysis of IKKα in tumors (Figures 4A and F). IKKα was mainly located in the basal cells, where the K5 promoter directs the transgene expression although it was also detected in the suprabasal layers (Figure 5A' inset). Differentiation markers such as the keratins K1/K10 are expressed at higher levels in WT tumors than in Tg tumors in both protocols of skin carcinogenesis (Figure 5B, B' and data not shown). K13, a keratin characteristic of internal stratified squamous epithelia which is aberrantly expressed in skin tumors (Figure 5C), indicating malignancy [49], was rarely expressed in WT tumors while it was extensively expressed in the K5-IKKα tumors (Figure 5C'). Low expression of keratins K1/K10 and elevated expression of K13 indicate a worse prognosis of tumors that express elevated levels of IKKα. Maspin expression was analyzed and found that it was lower in the K5-IKKα tumors (Figure 5D, D'). Panels A–D' in Figure 5 show staining of DMBA/TPA tumors although similar results were found in the Tg.AC tumors staining (data not shown).

#### Induction of twist and delocalized integrin-α6 expression in tumors developed in K5-IKKα Tg mice

We analyzed other markers of tumor progression, such as the expression of integrin-α6. In benign tumors, integrin-α6 is expressed by basal keratinocytes; however in malignant tumors it is also expressed in suprabasal layers [40], [48]. We found that tumors developed in WT mice in both carcinogenesis protocols have a basal staining of integrin-α6 (Figure 5E); by contrast, tumors from K5-IKKα mice exhibit basal as well as delocalized suprabasal expression of integrin-α6 (Figure 5E').

Another marker of tumor malignancy is Twist, which is expressed in embryonic development and silenced in the adulthood. However, it is induced in malignant tumors and is associated with metastasis [36]. We found that while WT tumors do not express Twist (Figure 5F), it is highly induced in K5-IKKα tumors obtained by both protocols of carcinogenesis (Figure 5F'). It is detected in the basal layer of the epidermis, where the K5 promoter directs the expression of the IKKα transgene (Figure 5F').

As increased integrin-α6 expression is usually accompanied by increased proliferation [46], we next analyzed tumor cell proliferation, measured as BrdU incorporation, and found higher number of proliferating cells in the K5-IKKα tumors (Figure 5G, G'). However, the size of IKKα and WT tumors showed no significant differences; therefore, we examined the apoptosis in both types of tumors and found that WT papillomas exhibited low number of apoptotic cells (measured by cleaved-Caspase 3 immunostaining; Figure 5H). By contrast, the number of apoptotic cells in IKKα tumors was markedly higher (Figure 5H'). Nevertheless, the rate of proliferation is greater than that of apoptosis in transgenic tumors and these differences alone would not fully explain the similar size reached by both types of tumors. Panels E–H' in Figure 5 show staining of DMBA/TPA tumors although similar results were found in the Tg.AC tumors staining (data not shown). Altogether these results suggest that skin tumors overexpressing IKKα in the basal layer of the epidermis have a malignant potential due at least in part to the induction of Twist expression and the suprabasal expression of integrin-α6.

## Discussion

We have found that the overexpression of IKKα in the epidermis of K5-IKKα mice causes several molecular alterations, such as increased cyclin D1 expression, delocalized suprabasal integrin-α6 expression and downregulation of the tumor suppressor maspin. These proteins are important for cancer development and progression, suggesting that the skin of these Tg mice could develop more aggressive lesions when subjected to skin injuries than WT skin. We have proved that in fact, after applying proliferative stimuli (such as TPA) in back skin, K5-IKKα mice develop epidermal atypia with loss of tissue architecture, being these pathological changes considered as preneoplastic signals. TPA also induces inflammation; the inflammatory response found both in WT and Tg mice following TPA treatment was similar, indicating that the alterations found in skin of Tg mice after TPA application are unlikely due to the proinflammatory activity of this agent. We have also found that in carcinogenesis assays Tg mice develop invasive tumors with higher malignant potential than the benign tumors developed in WT mice.

The first anomaly detected in the epidermis of the Tg K5-IKKα mice was an enhanced proliferation of basal keratinocytes that seems to be the consequence of both enhanced cyclin D1 expression and increased (and delocalized) integrin-α6 expression. Epidermal suprabasal integrin-α6 expression has been correlated with high proliferative activity in the basal layer of the epidermis, without occurrence of abnormal terminal differentiation or inflammation [46]. Our results are in line with these observations, since we find in K5-IKKα Tg mice increased proliferation of keratinocytes without inflammation or appreciable alterations in early or terminal differentiation. While our data are the first description of regulation of integrin-α6 by IKKα, the regulation of the expression of other adhesion proteins by IKKα has been previously reported: Changes in E-cadherin, desmoglein 3, claudin and occludin expression levels as a consequence of changes in IKKα expression have been described [5], [11], [50]. The induction of cyclin D1 expression by IKKα has also been previously reported [51]; in addition, we have also found increased cyclin D1 expression in human keratinocytes overexpressing IKKα (Alameda et al, unpublished data).

The suprabasal integrin-α6 expression found both in epidermis and skin tumors of Tg mice could also cause the increased skin malignancy of K5-IKKα tumors, as inappropriate suprabasal integrin-α6β4 expression in epidermis correlates with high risk of cancer progression [39]. In this regard, it is interesting to note that the delocalized integrin-α6 expression in papillomas is an early predictive marker for the identification of benign squamous tumors at high risk of malignant progression [40].

An interesting finding of this study has been the discovery of IKKα as negative regulator of the tumor suppressor maspin in the skin, since downregulation of this protein has been related to tumor progression and metastasis [17], [20], [21]. The downregulation of maspin by IKKα in epidermis has not been described before and we have confirmed this result in other different transgenic mice expressing distinct IKKα constructs (Alameda et al, unpublished results). Additionally, we have found that Twist expression which is silenced in adult tissues is induced in K5-IKKα tumors. In our experience searching in mouse skin cancer we have analyzed by immunohistochemistry more than 80 skin tumors obtained by both protocols of chemical carcinogenesis, and wee have never found before the induction of Twist expression in keratinocytes. Twist induction could be another plausible reason for the increased aggressiveness of the K5-IKKα tumors (developed in Tg.AC and DMBA-treated mice, carrying an activated Ha-*ras*), since recent findings show that in the presence of aberrant mitogenic signalling, such as Ha-*ras* activation, reactivation of Twist promotes the transition from a premalignant to a malignant stage by inactivation of innate failsafe programs [38]. It is well known that Twist is reactivated in different types of tumors and it is considered to play a key role in the development and progression of human cancer, being associated with advanced tumor stage and poor prognosis in rhabdomyosarcoma, gastric carcinoma, melanoma, glioma, liver carcinoma and breast, prostate, bladder and pancreatic cancer [37]; [52], [53]; [36], [54]. The regulation of Twist expression by IKKα has been previously noted, in other context, by Takeda et al., who described the downregulation of *twist* in IKKα−/− embryos [7].

During the last years several evidences have been reported indicating that IKKα functions as an oncogenic molecule. For instance, IKKα phosphorylates important molecules of signalling cascades (β-catenin, estrogen receptor-α transcriptional factor) which through induction of cyclin D1 and/or c-Myc expression enhances tumor proliferation [51], [55], [56], [57]. Recently, a nuclear function of IKKα has been implicated in tumor progression: [58], [59], [19], [60]. In this regard and in agreement with the data obtained in this study, it is interesting to mention the role of nuclear IKKα in promoting cancer through inhibition of maspin expression in pancreatic cancer [17], [35]. Other evidences of the implication of IKKα in tumor promotion are that IKKα is induced by different proangiogenic agents such as TPA, UV radiation and Ets1 and that IKKα itself promotes angiogenesis and stimulates tumoral growth [61]. In this context, it is worth to note that IKKα KO mice have impaired angiogenesis [8]. In agreement with all these data, there is an increasing number of studies reporting the relationship of IKKα signaling to the development of different types of neoplasias: breast cancer [14]; hepatocarcinomas [15]; prostate cancer [17], and colorectal cancer [18]. Our group has also found an increase in the malignancy of skin tumors arising after injection of tumor epidermal cells overexpressing IKKα into nude mice [5].

On the other hand, other studies indicate a role for IKKα as a tumor suppressor in skin cancer. Therefore, Loricrin-IKKα transgenic mice that overexpress IKKα in the suprabasal terminally differentiated cells, which are mitotically inactive and committed to shed, develop less tumors in skin carcinogenesis experiments than WT mice [4]. However, this approach is not comparable to ours because we have targeted IKKα to basal keratinocytes, which are mitotically active cells. This is an important difference, because the tumorigenic properties of skin tumors strongly depend on the cell type targeted being the expression of a potential tumoral promoter more harmful in basal cells than in terminally differentiated cells [62]. Other studies on the role of IKKα in skin tumorigenesis have been performed in IKKα+/− mice and show that these animals develop more carcinomas with a lower latency period [13]. However, these mice are defective in IKK expression in both epidermis and dermis, and increasing evidences support the contribution of the tumor stroma to some of the most malignant characteristics of epithelial tumors [63]; therefore through this approach it is not possible to discern the role that the expression of IKKα specifically in keratinocytes plays for skin carcinogenesis. A different approach to this study would be the use of conditional knockout mice lacking IKKα specifically in keratinocytes. These mice have been generated by two different groups and the skin phenotypes obtained are completely different: while one model exhibits an hyperplastic skin with absence of terminal differentiation [12], the other shows a nearly normal skin with terminal differentiation and no signs of hyperplasia [11] being the reasons for this discrepancy not understood. Therefore, although skin carcinogenesis assays showing increased tumorigenesis in the IKKα conditional mice exhibiting a skin phenotype have been reported [12], the absence of the same experiments in the other IKKα conditional mice model casts doubts on the conclusiveness of the results.

Taking into account the different results published, it seems that the role that IKKα plays in carcinogenesis could depend on the type of tumor, the cell targeted in each tumor and the strain of mice employed in the studies. Our present study supposes a different approach for study the role of IKKα in skin carcinogenesis, targeting IKKα to the basal keratinocytes of the epidermis. Our results showing the increase in the malignant potential of skin tumors developed *in vivo*, in transgenic mice overexpressing IKKα in keratinocytes, are in line and strengthen our previous findings showing the enhanced aggressiveness of skin tumors arising after injection of tumor epidermal cells overexpressing IKKα into nude mice [5]. We have found that increased IKKα expression levels in the basal layer of the epidermis and ORS of the hair follicles of transgenic mice leads to alterations that originate lesions of higher malignant potential than those developed in WT mice when subjected to aberrant mitogenic stimuli. We have found that the altered expression of cyclin D1, maspin and integrin-α6 in skin of transgenic mice provide, at least in part, the molecular bases of the increase in the malignant potential of carcinomas originated in skin of K5-IKKα Tg mice.

## Supporting Information

Figure S1 Graphical representation of the number of tumors developed in WT and Tg-K5-IKKα mice in the two skin carcinogenesis approaches. (**A**) K5-IKKα and WT 9-week-old mice (9 animals respectively) were subjected to DMBA/TPA carcinogenesis assay. Tumors were traced until week 26, when they were collected. (**B**) Double transgenic K5-IKKα-TgAC and WT-TgAC 9-week-old mice (11 animals respectively) were treated twice weekly with topical applications of TPA. Tumors were traced until week 30, when they were collected. No differences were found between number of tumors developed in WT and Tg animals in both approaches.
(TIF)
