# Supplementary figures and images for: Increased IKKα Expression in the Basal Layer of the Epidermis of Transgenic Mice Enhances the Malignant Potential of Skin Tumors
Source: PLoS One. 2011 Jul 6;6(7):e21984. doi: 10.1371/journal.pone.0021984 (PMC3130791; doi:10.1371/journal.pone.0021984)

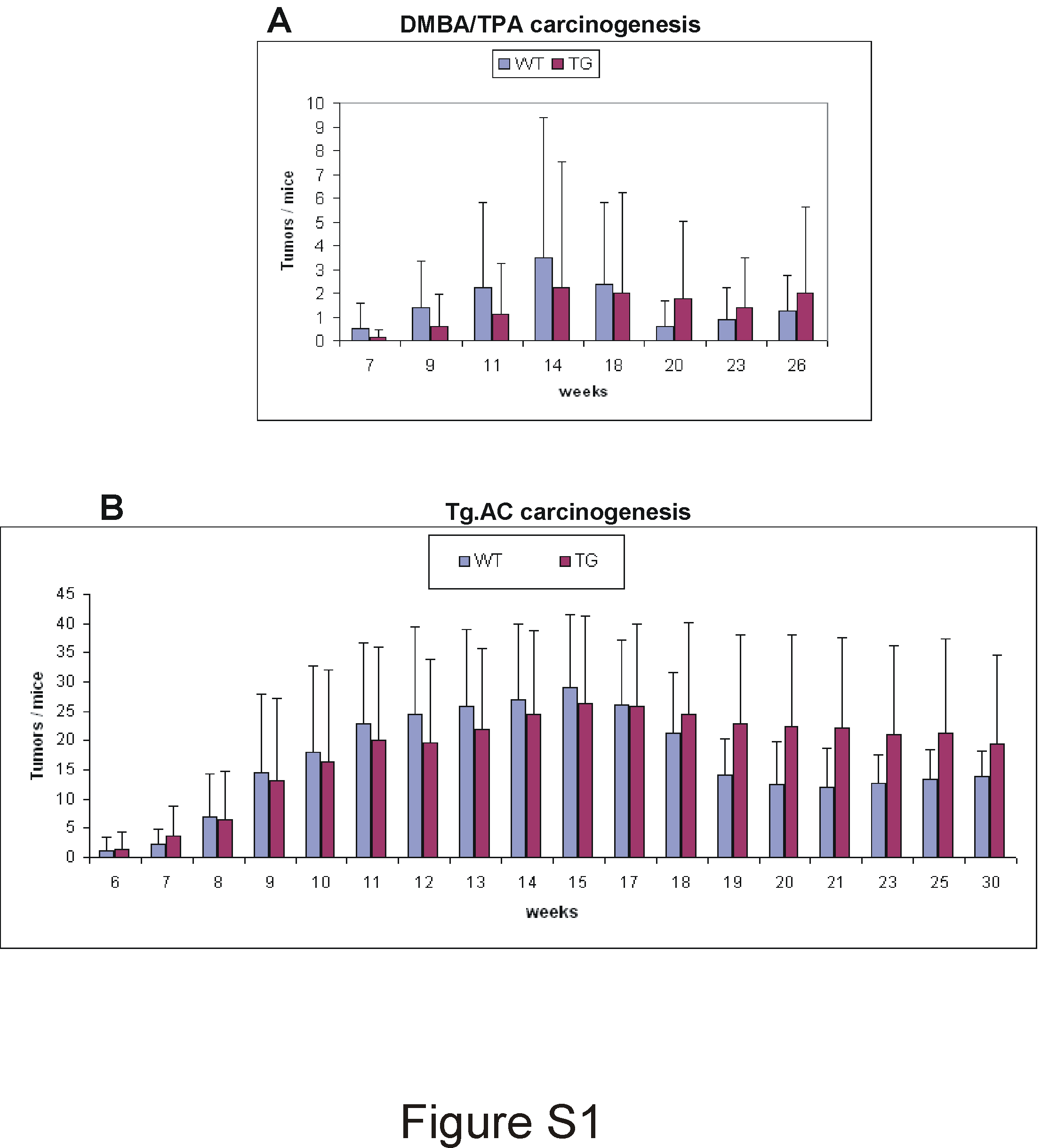

Supplement: Figure S1 — Graphical representation of the number of tumors developed in WT and Tg-K5-IKKα mice in the two skin carcinogenesis approaches. (A) K5-IKKα and WT 9-week-old mice (9 animals respectively) were subjected to DMBA/TPA carcinogenesis assay. Tumors were traced until week 26, when they were collected. (B) Double transgenic K5-IKKα-TgAC and WT-TgAC 9-week-old mice (11 animals respectively) were treated twice weekly with topical applications of TPA. Tumors were traced until week 30, when they were collected. No differences were found between number of tumors developed in WT and Tg animals in both approaches. (TIF) [file pone.0021984.s001.tif]
